# Aerobic and resistance training enhances endothelial progenitor cell function via upregulation of caveolin-1 in mice with type 2 diabetes

**DOI:** 10.1186/s13287-019-1527-z

**Published:** 2020-01-03

**Authors:** Lu Zhai, Yuhua Liu, Wenpiao Zhao, Qingyun Chen, Tao Guo, Wei Wei, Zhuchun Luo, Yanfeng Huang, Cui Ma, Feng Huang, Xia Dai

**Affiliations:** 1 Department of Endocrinology, The First Affiliated Hospital of Guangxi Medical University, Nanning, 530021 China; 2 Department of Nursing, Guangxi JiangBin Hospital, Nanning, 530021 China; 3 Department of Cardiology, The First Affiliated Hospital of Guangxi Medical University, Nanning, 530021 China; 4 Department of Gastroenterology, The First Affiliated Hospital of Guangxi Medical University, Nanning, 530021 China; 5 Department of Internal Medicine, The First Affiliated Hospital of Guangxi Medical University, Nanning, 530021 China

**Keywords:** Endothelial progenitor cell, Type 2 diabetes, Aerobic and resistance training, Caveolin-1

## Abstract

**Background:**

To explore the effect of aerobic training (AT), resistance training (RT) or a combination of AT and RT (AT+RT) on the function of endothelial progenitor cells (EPCs) in mice with type 2 diabetes and the potential effective mechanisms

**Methods:**

Eight-week-old db/db male mice were used as type 2 diabetic animal models in this study. Mice were randomly assigned to the control group (*n* = 5), AT group (*n* = 5), RT group (*n* = 5) and AT+RT group (*n* = 5). Mice in the control group remained sedentary with no specific training requirement. Mice were motivated to perform AT, RT or AT+RT by a gentle pat on their body for 3 or 4 days/week for 14 days. AT was performed by treadmill running, RT was performed by ladder climbing and AT+RT involved both AT and RT. Bone-derived EPCs were isolated after 14 days of the intervention. EPC expression of CD31, CD34, CD133, CD144 and VEGFR2 was detected by immunofluorescence staining. Fluorescence detection was performed on attached mononuclear cells to detect double-positive EPCs. We then explored the effect of caveolin-1 knockdown (lentiviral vector with caveolin-1-siRNA) on the proliferation and adherence of EPCs and the concentration of caveolin-1 and PI3K/AKT via western blot analyses.

**Results:**

Compared to the mice in the control group, the mice in the AT, RT and AT+RT groups presented significant increases in proliferation and adherence after 14 days of intervention. AT+RT induced an increase in EPC adherence, which was greater than that of the control, RT and AT groups. Caveolin-1 knockdown inhibited the EPC proliferative and adherent abilities. The AT+RT group showed higher levels of caveolin-1 and p-AKT than the control group, but these changes were decreased by caveolin-1-siRNA transfection.

**Conclusion:**

Combined AT and RT is an effective way to improve EPC function through upregulation of caveolin-1 in mice with type 2 diabetes.

## Background

Bone marrow-derived endothelial progenitor cells (EPCs) can self-proliferate to increase their cell number and adhere to injured vessels to maintain the integrity of the vascular system, especially via endothelial regeneration. Low EPC levels have been shown to be an independent risk factor for future cardiovascular events and overall mortality in different populations, even after adjustment for diabetes, age and blood glucose. Compared to people without diabetes, those with diabetes have fewer EPCs and impaired EPC function, which may contribute to an increased risk of cardiovascular disease and other vascular complications [1, 2].

Aerobic exercise was reported to improve the proliferation, migration and angiogenesis of EPCs in healthy individuals and patients with metabolic syndrome, congestive heart failure, coronary heart disease and prediabetes [3–7], resulting in maintenance of the endothelial monolayer integrity. The integrity of the endothelial monolayer may play a role in preventing thrombotic complications and atherosclerotic lesion development [8]. Resistance exercise was also reported to improve metabolic profiles in patients with diabetes. A systematic review noted that resistance exercise may be more feasible than aerobic exercise for non-alcoholic fatty liver disease patients with poor cardiorespiratory fitness or for those who cannot tolerate or participate in aerobic exercise [9]. Recently, resistance exercise and combined aerobic and resistance exercise have received increased attention due to their ability to increase the number of EPCs in the peripheral circulation of healthy people [10–13].

The underlying mechanisms of exercise-induced EPC function have not been identified; however, caveolin-1 was found to be abundant in endothelial cells. A previous study showed that caveolin-1 plays a central role in EPC mobilization and homing in stromal cell-derived factor 1 (SDF-1)-driven post-ischaemic vasculogenesis [14]. The increase in EPC function stimulated by PI3K/AKT could be decreased by a PI3K inhibitor [15, 16].

Since little is currently known about the effects of exercise on EPC function, we aimed to explore the effects of aerobic training (AT), resistance training (RT) or a combination of aerobic and resistance training (AT+RT) on the function of EPCs and the protein expression of caveolin-1, PI3K and AKT in mice with type 2 diabetes. We aimed to provide a helpful reference for the selection of exercise methods to prevent diabetes-related cardiovascular diseases.

## Methods

All animal procedures were performed at Guangxi Medical University. Ethical approval was granted by the Guangxi Medical University Institutional Animal Care and Use Committee. Eight-week-old male type 2 diabetic db/db (BKS-Dock Lepr^em2Cd479^/Nju) littermates [17] were obtained from the Nanjing Biomedical Research Institute of Nanjing University, Nanjing, China. The mice were randomly divided into the control group, AT group, RT group and AT+RT group (*n* = 5/each group). During the 14 days of the intervention, a tail blood sample was obtained for glucose measurement with a glucose metre (Johnson & Johnson, Milpitas, CA, USA) before starting the experiment and 24 h after training, which was repeated every week. Type 2 diabetes was diagnosed when the blood glucose level of a mouse was above 11.1 mmol/l twice. The db/db mice had mutant copies of the gene encoding the leptin receptor and exhibited an obese type 2 diabetic phenotype when they were 6 weeks old. Mice were euthanized with intraperitoneal administration of 100 mg/kg sodium pentobarbital, followed by isolation of the bone marrow-derived EPCs.

### Exercise training

Four or five mice were housed per cage, fed ordinary chow and maintained under a 12-h light/12-h dark cycle. The mice in the control group were maintained in a sedentary state, and the mice in the exercise groups performed AT, RT or AT+RT.

AT was performed on a treadmill (WDW-1, Beijing North Rui Future Analytical Instrument Co., Ltd., Beijing, China) specifically designed for mice. The mice in the AT group were trained for a total of 14 days over 4 weeks (the mice ran 3–4 days a week). Mice were initially trained at a speed of 10 m/min for 10 min 3 days/week in the first week. From the second week, the exercising mice performed AT for 50 min at a speed of 13–17 m/min 3 days in the second week and 4 days in the next 2 weeks [18]. All mice ran at the same speed, which increased from 13 to 17 m/min when the mice were able to run for 50 min without rest. The running speed and distance run on the treadmill could be adjusted by the researchers, and thus, we could control the running protocol and training times of the mice.

**Fig. 1:**
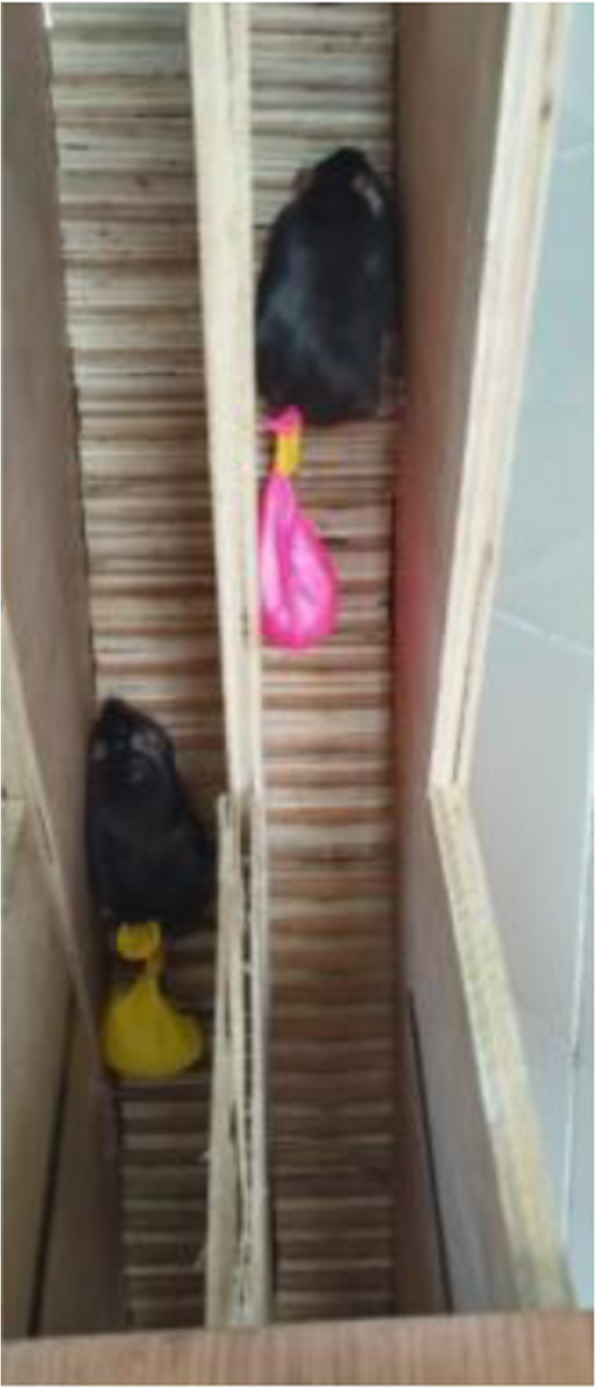
Image of the ladder. The mice in the AT+RT group performed AT following the protocol of the AT group on Monday, Wednesday, Friday and Sunday and performed RT following the protocol of the RT group on Tuesday, Thursday, Saturday and Sunday. The exercise protocol was discontinued 48 h before sacrifice

The mice in the RT group climbed a 1-m custom-made ladder composed of a 2-cm grid inclined at 85° (see Fig. 1 below). The mice in the RT group were trained for a total of 14 days over 4 weeks (the mice ran 3–4 days a week). The mice climbed from the bottom of the ladder to the top while the appropriate weights were attached to their tails. The weight was 50% of the mouse’s body weight in the first week and increased gradually throughout the training period. The mice were motivated to climb the ladder by a gentle pat on their body until they finished three sets of five repetitions of climbing for 3 or 4 days/week for 14 days. The mice were allowed to rest for 1 min between repetitions and 2 min between the sets [18].

### Isolation and culture of bone marrow-derived EPCs

EPCs were isolated when the exercise protocol was discontinued for 48 h. Five mice from each group (20 mice in total) were intraperitoneally euthanized with 100 mg/kg sodium pentobarbital in accordance with the rules of the Institutional Ethical Board for Experimental Procedures. The tibiae and femurs of the mice were bluntly dissected. Then, the medullar channels were flushed with 10 ml of phosphate-buffered saline (PBS, Beijing Solarbio Science & Technology Co., Ltd., Beijing, China). The cell suspensions were collected by filtration and added to the lymphocyte separating solution (Beijing Solarbio Science & Technology Co., Ltd., Beijing, China) at a ratio of 1:1. The cell suspensions were centrifuged at 400*g* for 30 min. The cloudy layer of cells was collected and mixed with endothelial cell basal medium-2 (EBM-2; Lonza Group, Ltd., Basel, Switzerland) and then centrifuged at 400*g* for 30 min. The precipitation was subsequently resuspended in EBM-2 containing 10% foetal bovine serum (FBS; Gibco; Thermo Fisher Scientific, Inc., Waltham, MA, USA) and vascular endothelial growth factor (VEGF; R&D Systems, Shanghai Sixin Biotechnology Co., Ltd., Shanghai, China). Cells were counted under a microscope and seeded in 25-cm^2^ cell culture bottles coated with human plasma fibronectin purified protein (FPP; R&D Systems, Shanghai Sixin Biotechnology Co., Ltd., Shanghai, China) at a concentration of 10^6^ cells/cm^2^. The cells were grown for 4 days. Then, the cells suspended in the medium were removed, and the cells adhering to the surface were cultured for another 3 days [19].

### Cellular staining

Fluorescent chemical detection of EPCs was performed on the attached mononuclear cells after 7 days of culture. Direct fluorescence staining was used to detect the dual binding of fluorescein isothiocyanate (FITC)-labelled *Ulex europaeus* agglutinin (UEA)-1 (Sigma Chemical Co., St. Louis, MO, USA) and 1,1-dioctadecyl-3,3,3,3-tetramethylindocarbocyanine (DiI)-labelled acetylated low-density lipoprotein (ac-LDL; Molecular Probes, Eugene, OR, USA). Cells were first incubated with ac-LDL (10 μg/ml) at 37 °C for 4–6 h and then fixed with 4% paraformaldehyde for 10 min. Subsequently, the washed cells were incubated with UEA-1 (10 μg/ml) for 1.5 h. After staining, the samples were observed under an inverted fluorescence microscope (IX71, Olympus Corporation, Shibuya, Tokyo, Japan) and further analysed by a laser scanning confocal microscope (FV1000, Olympus Corporation). The cells showing double-positive fluorescence were identified as differentiating EPCs. The EPC number was determined by counting the number of cells in five randomly selected high-power fields (× 200) via an inverted fluorescence microscope (IX71, Olympus Corporation, Shibuya, Tokyo, Japan). Each experiment was performed with three replicates to ensure the reliability of the data.

### Immunofluorescence staining analysis

The cells were washed with PBS, fixed with 3–4 ml of 4% paraformaldehyde for 30 min at 4 °C, washed with PBS three times and finally permeabilized with a mixture of 10% goat serum (Beijing Solarbio Science & Technology Co., Ltd., Beijing, China) and 0.5% Triton X-100 (Beijing Solarbio Science & Technology Co., Ltd., Beijing, China). The cells were immunofluorescently stained with CD31 (Proteintech Group, Inc., Chicago, USA), CD34 (Beijing Bioss Molecular Co., Ltd., Beijing, China), CD133 (Proteintech Group, Inc., Chicago, USA), CD144 (Beijing Bioss Molecular Co., Ltd., Beijing, China) and VEGFR2 (Proteintech Group, Inc., Chicago, USA) antibodies, which were diluted to 100 ml, overnight at 4 °C. The cells were then washed with PBS three times (5 min/time) and incubated with CoraLite 488-conjugated secondary antibody (Proteintech Group, Inc., Chicago, USA) and Alexa Fluor 594-conjugated secondary antibody (Proteintech Group, Inc., Chicago, USA) in the dark at room temperature for 2 h. The cell DNA was stained with 4′,6-diamidino-2-phenylindole (DAPI) (Beijing Solarbio Science & Technology Co., Ltd., Beijing, China). The cells were examined by fluorescence microscopy (IX71, Olympus Corporation, Shibuya, Tokyo, Japan).

### Proliferation assay

Suspended EPCs were plated on a collagen-coated 96-well plate (3.6–4.0 × 10^3^ cells/well) and cultured for 24 h. Subsequently, the suspended EPCs were incubated for another 4 h in the dark following the addition of 10 μl of CCK-8 solution (Dojindo Molecular Technologies, Inc., Kumamoto, Japan) in each well. Then, the plate was agitated for 10 s to prepare for optical density (OD) measurement, which was performed at an absorbance of 450 nm with a microplate reader (ELX800; BioTek Instruments, Inc., Winooski, VT, USA).

### Adherence assay

Suspended EPCs were plated on an FPP-coated 96-well plate (2 × 10^5^/ml) cultured for 24 h at 4 °C. Subsequently, the suspended EPCs were incubated with CCK-8 solution (Dojindo Molecular Technologies, Inc., Kumamoto, Japan) in each well for another 2 h in the dark. Then, the plate was agitated for 10 s to prepare for OD measurement, which was performed at an absorbance of 450 nm with a microplate reader (ELX800; BioTek Instruments, Inc., Winooski, VT, USA).

### Western blot analysis

After cultivation for 7 days, fractionation of EPCs was performed with RIPA lysis buffer (Beyotime Institute of Biotechnology, Shanghai, China), which contained 1% phenylmethanesulfonyl fluoride and phosphatase inhibitor (Beijing Solarbio Science & Technology Co., Ltd., Beijing, China). The protein concentrations were determined by the bicinchoninic acid method according to the manufacturer’s instructions (Beyotime Institute of Biotechnology, Shanghai, China). Total protein was separated via 10% SDS-PAGE, transferred to a polyvinylidene difluoride membrane (Millipore) and blocked with 5% skim milk. Primary antibodies against the following proteins were used for western blotting: caveolin-1 (Cell Signaling Technology), total PI3K p85 (t-p-PI3Kp85, Abcam), phosphorylated PI3K p85 (p-PI3Kp85, Abcam), total AKT (t-AKT, Cell Signaling Technology) and phosphorylated AKT (p-AKT, Cell Signaling Technology). The membranes were washed with TBST buffer (Beijing Leagene Biotechnology Co., Ltd.) and probed with HRP-conjugated secondary antibodies (goat anti-rabbit or goat anti-mouse IgG). Protein band and densitometric analyses were performed using enhanced chemiluminescence (LI-COR Biosciences) to quantify the protein concentrations.

### Knockdown of caveolin-1

EPCs were plated on a 6-well plate (1 × 10^5^ cells/well) and cultured for 24 h until the cell confluence was approximately 70–80%. Subsequently, the suspended EPCs were divided into the Lenti-caveolin-1 siRNA silence group (caveolin-1 siRNA) and the Lenti-caveolin-1 siRNA-Mut group (caveolin-1-Mut). The intended meaning of the sentence“EPCs in the caveolin-1 siRNA were incubated a lentiviral vector expressing a caveolin-1 small interfering RNA (si) RNA (Shanghai GeneChem Co., Ltd) with a multiplicity of infection (MOI) of 50. EPCs in caveolin-1-Mut were transfected with a lentiviral vector expressing a caveolin-1 non-targeting RNA (Shanghai GeneChem Co., Ltd). Polybrene with 8 μg/ml was added to accelerate transfection. Transfection was performed at 37 °C for 6 h. Subsequently, the cells were photographed after culture for another 72 h.

### Statistical analysis

All variables were tested for normality by the Kolmogorov-Smirnov test. Levene’s test was performed to test for equality of variances. If the variables were homogeneous, one-way ANOVA was used for the analysis of the different variables among the groups, and the Tukey test was used for post hoc analyses; otherwise, the Welch test was chosen for alternative between-group comparisons, and the Tamhane test was used for post hoc analyses. Data are shown as the mean ± SE. *P* < 0.05 (two-tailed) was defined as significant. Data were analysed using SPSS software 22.0 (SPSS Inc., Chicago, IL, USA).

## Results

### Characterization of EPCs

**Fig. 2:**
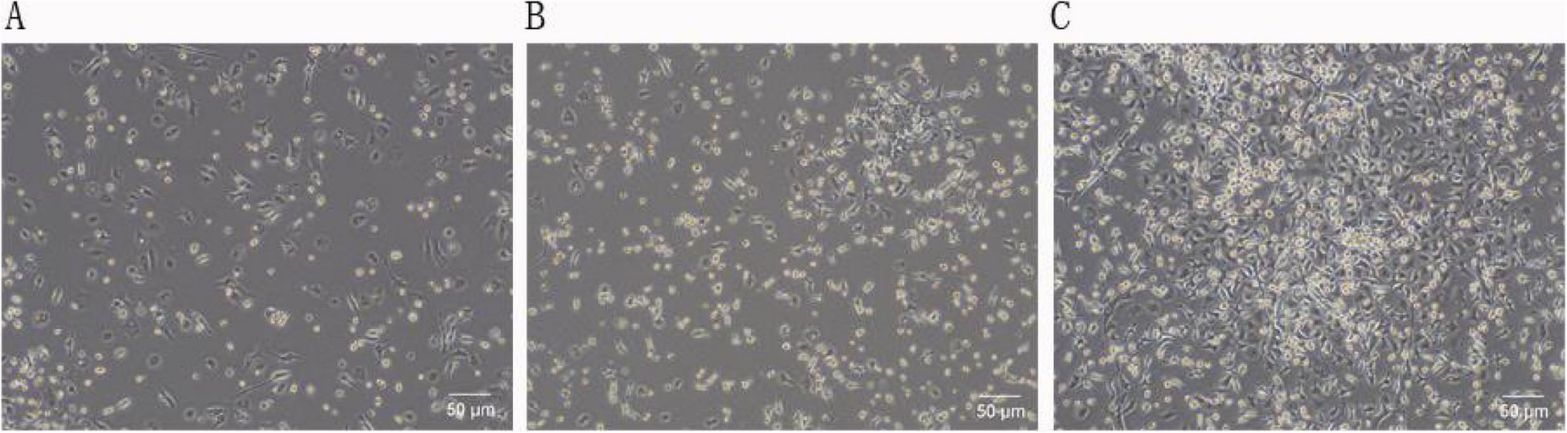
Characterization of EPCs of mice isolated from the control group growing in vitro (magnification × 100). **a** Round or oval EPCs on the 4th day. **b** Spindle-shaped or round EPCs on the 8th day. **c** EPCs covering the bottom, similar to “stone pavement”, on the 14th day. After 7 days of growth, the bone marrow-derived EPCs from mice were characterized as adherent cells that showed DiI-ac-LDL uptake and lectin binding via laser scanning confocal microscopy (Fig. 3). A total of 73.50 ± 7.47% of the adherent cells showed DiI-ac-LDL uptake and lectin binding after growing for 7 days. In addition, these findings were confirmed by the expression of the well-established cell surface markers CD31, CD34, CD133, CD144 and VEGFR2, which were detected in 91.2 ± 3.6%, 95.3 ± 2.6%, 94.8 ± 3.2%, 93.1 ± 4.2% and 95.8 ± 2.5% of all cells by immunofluorescence staining (see details in Fig. 4)

**Fig. 3:**
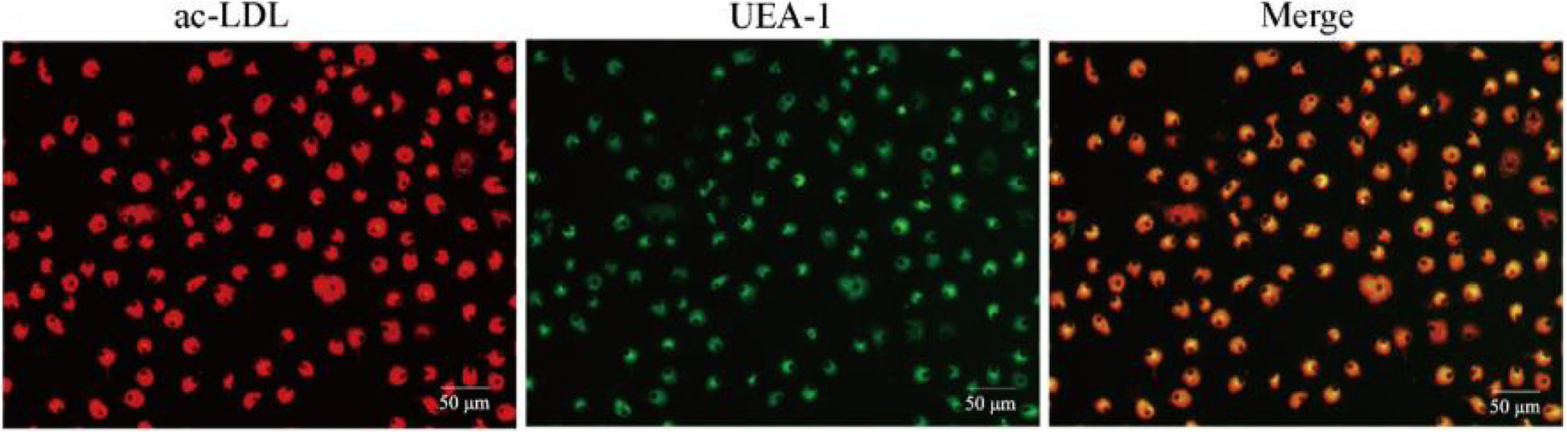
Bone marrow-derived EPCs of mice isolated from the control group after 7 days of growth. DiI-ac-LDL uptake (red) and lectin binding (green) of adherent cells were identified via laser scanning confocal microscopy. Double-positive cells appearing yellow in the overlay were identified as differentiating EPCs (magnification × 200)

**Fig. 4:**
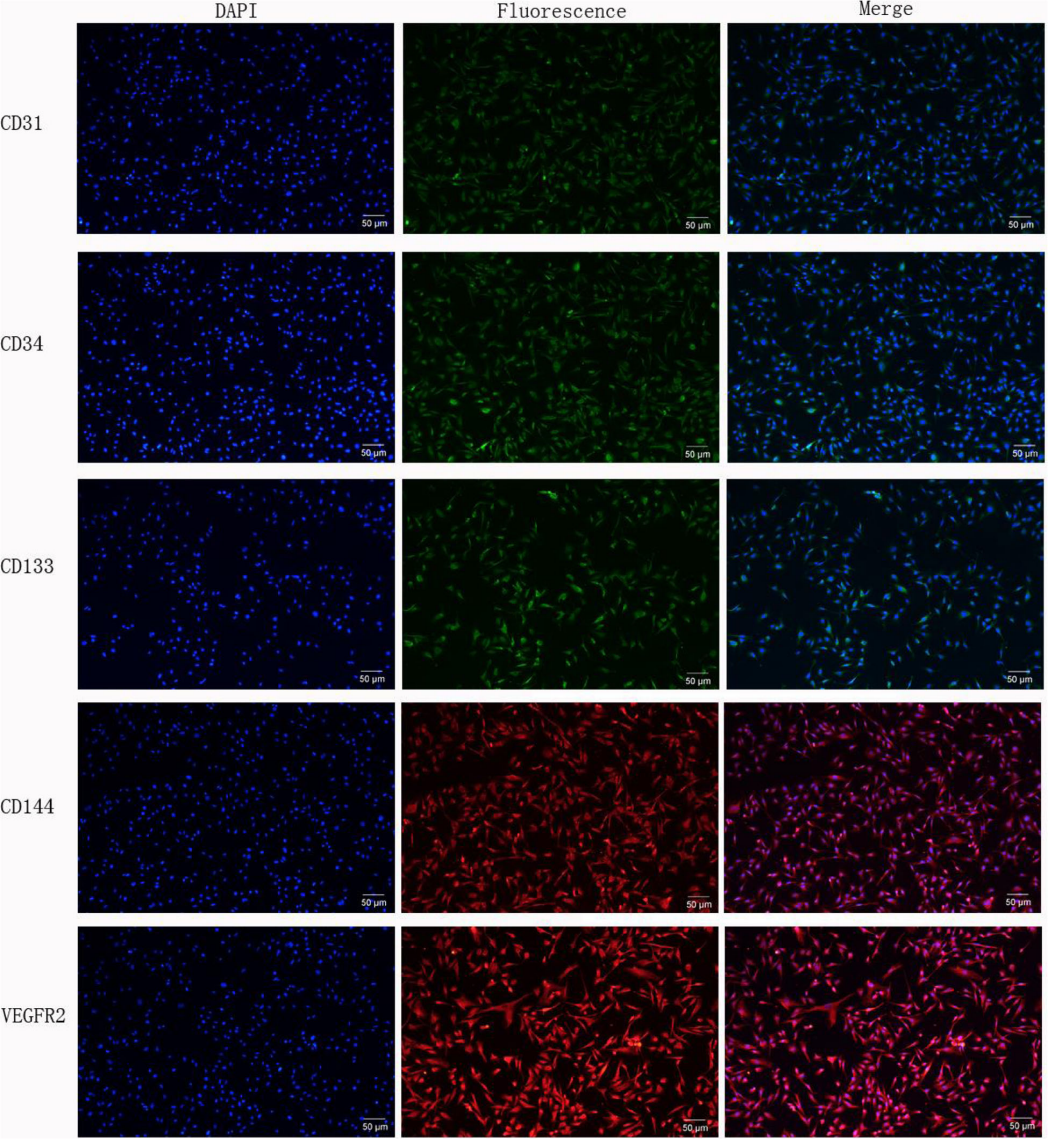
Immunofluorescence staining of CD31, CD34, CD133, CD144 and VEGFR2 (magnification × 100). Blue shows the nuclei of the EPCs via DAPI staining; the merged figures indicate cells with immunofluorescence staining of CD31, CD34, CD133, CD144 and VEGFR2, which accounted for 91.2 ± 3.6%, 95.3 ± 2.6%, 94.8 ± 3.2%, 93.1 ± 4.2% and 95.8 ± 2.5% of all cells, respectively

As shown in Fig. 2, after 4 days of growth in vitro, most of the EPCs were round or oval, and a few were spindle-shaped. On the 8th day, the EPCs were mostly spindle-shaped and round. On the 14th day, the cells grew well and adhered to the bottom of the culture bottle, similar to “stone pavement” (Figs. 3 and 4).

### Changes in glucose between the groups with different interventions

**Fig. 5:**
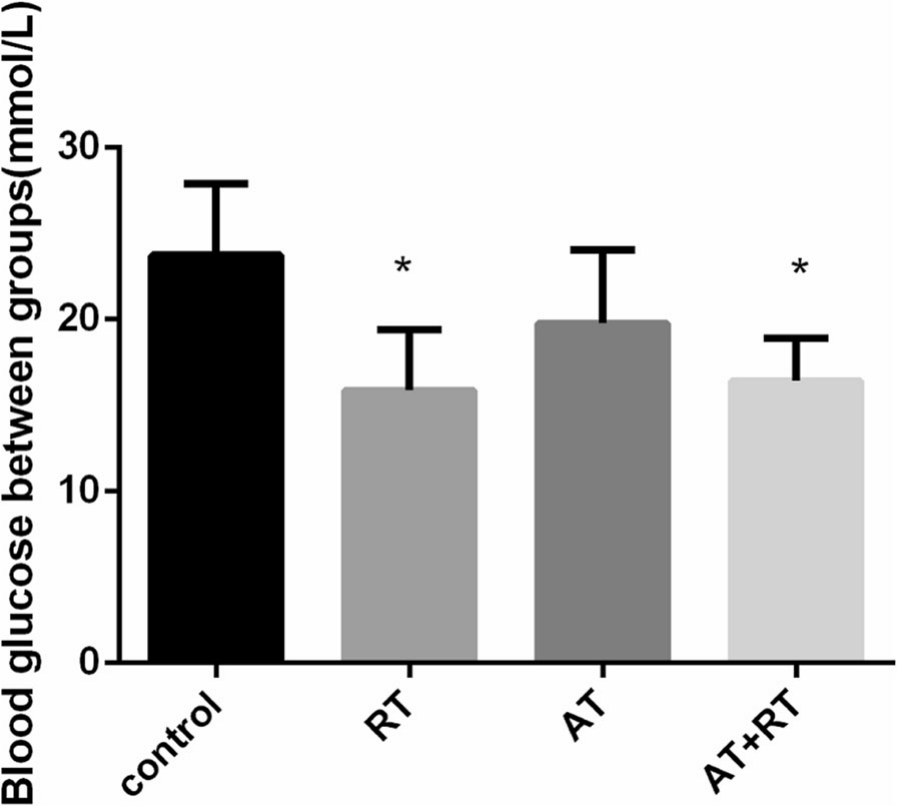
Blood glucose of mice following the exercise intervention. Note: control represents the control group, RT represents the resistance training group, AT represents the aerobic training group and AT+RT represents combined aerobic and resistance training. *P < 0.05 vs the control group

After interventions for 14 days, the blood glucose of mice in the RT and AT+RT groups was significantly lower than that in the control group (see Fig. 5 for more details).

### Proliferative ability

**Fig. 6:**
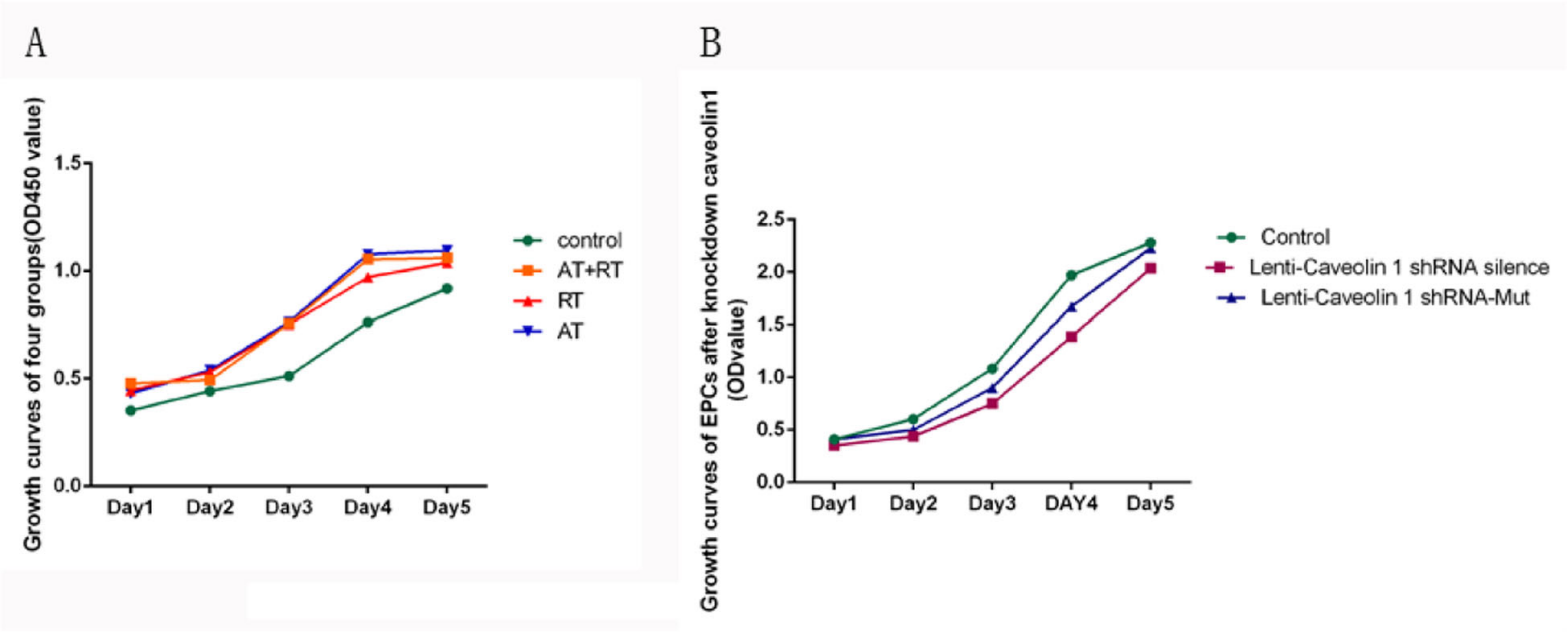
Growth curve of the EPCs. **a** Growth curves of the EPCs after 14 days of intervention. **b** Growth curves of the EPCs after knockdown of caveolin-1. AT+RT, combined aerobic and resistance training; RT, resistance training; AT, aerobic training

As shown in Fig. 6a, cell proliferation increased in the AT, RT and AT+RT groups compared with the control group after 14 days of intervention. The proliferative activities of the EPCs were significantly lower in the control group than in the AT group (vs control: 95% CI − 0.49 to approximately − 0.14, *P* < 0.001), the RT group (vs control: 95% CI − 0.38 to approximately − 0.04, *P* = 0.015) and the AT+RT group (vs control: 95% CI − 0.46 to approximately − 0.12, *P* = 0.001). There were no significant differences in the proliferative activity among the AT, RT and AT+RT groups. Knockdown of caveolin-1 retarded EPC growth. An increased proliferative capability was found in the control group compared to the caveolin-1-siRNA-silenced group (vs control: 95% CI 0.19~0.30, *P* < 0.001), but no difference was found between the control group and the negative control group (the caveolin-1-siRNA Mut group).

### Adherent ability

Exercise induced EPC adherence during the intervention. Compared with the control group, the AT+RT group showed significant increases in EPC adherence (vs control: 95% CI − 0.39 to approximately − 0.12, *P* < 0.001), and this increase was more notable in the AT+RT group than in the AT (vs AT+RT: 95% CI 0.03~0.30, *P* = 0.008) or RT (vs AT+RT: 95% CI 0.04~0.32, *P* = 0.017) groups. Caveolin-1 deficiency inhibited EPC adherence, which was 30% lower than that of the control group (vs control: 95% CI 0.17~0.51, *P* = 0.002).

### The concentration of caveolin-1 and PI3K/AKT determined by western blot analysis

**Fig. 7:**
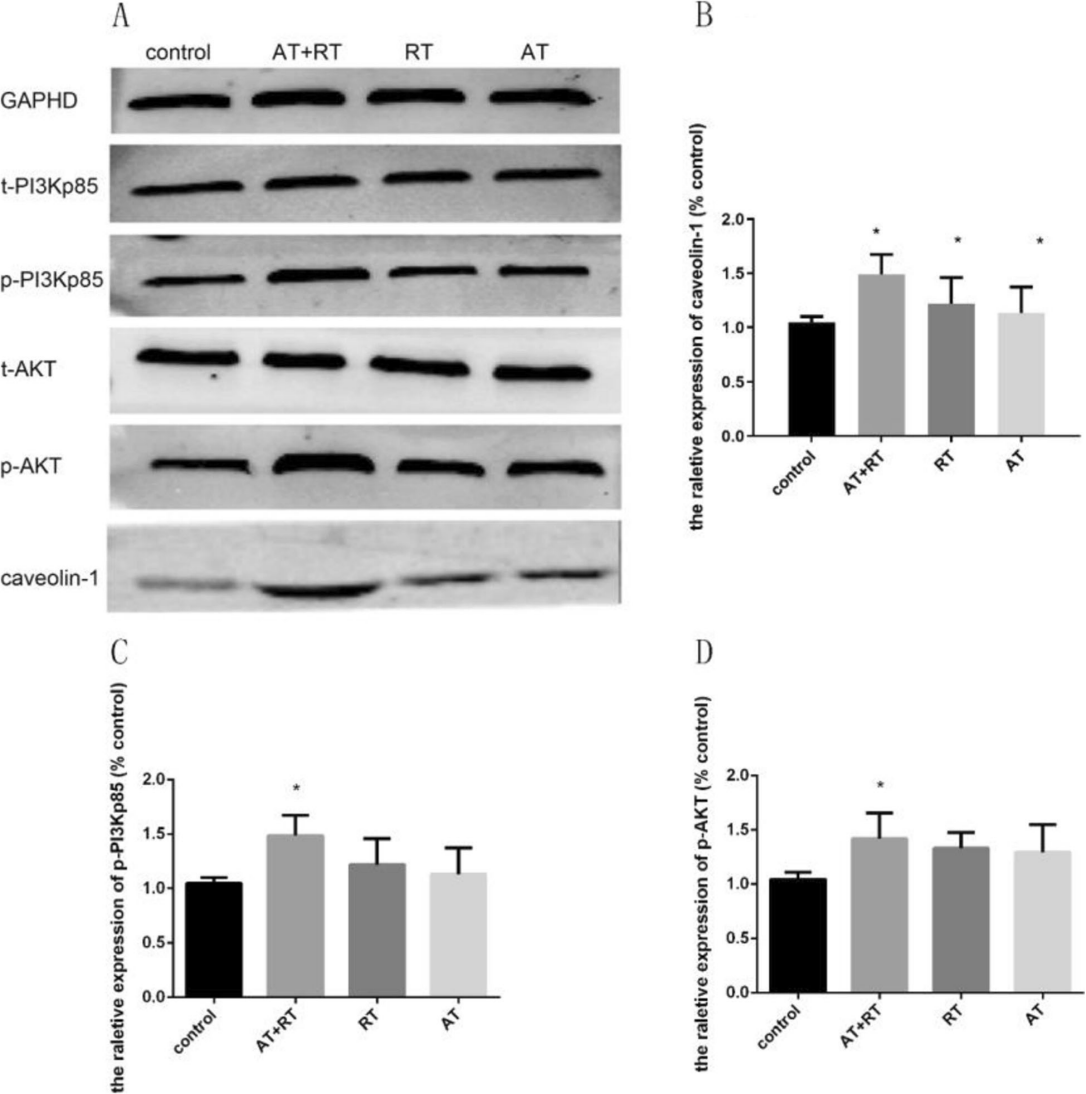
The caveolin-1 and PI3K/AKT protein levels in all groups after intervention. **a** The protein bands after 14 days of intervention. **b** Comparison of the caveolin-1 concentrations among the four groups. **c** Comparison of the p-PI3Kp85 concentrations among the four groups. **d** Comparison of the p-AKT concentrations among the four groups. p-PI3K, phosphorylated PI3K; p-AKT, phosphorylated AKT; AT, aerobic training; RT, resistance training; AT+RT, combination of aerobic and resistance training. *P < 0.05 vs the control group

**Fig. 8:**
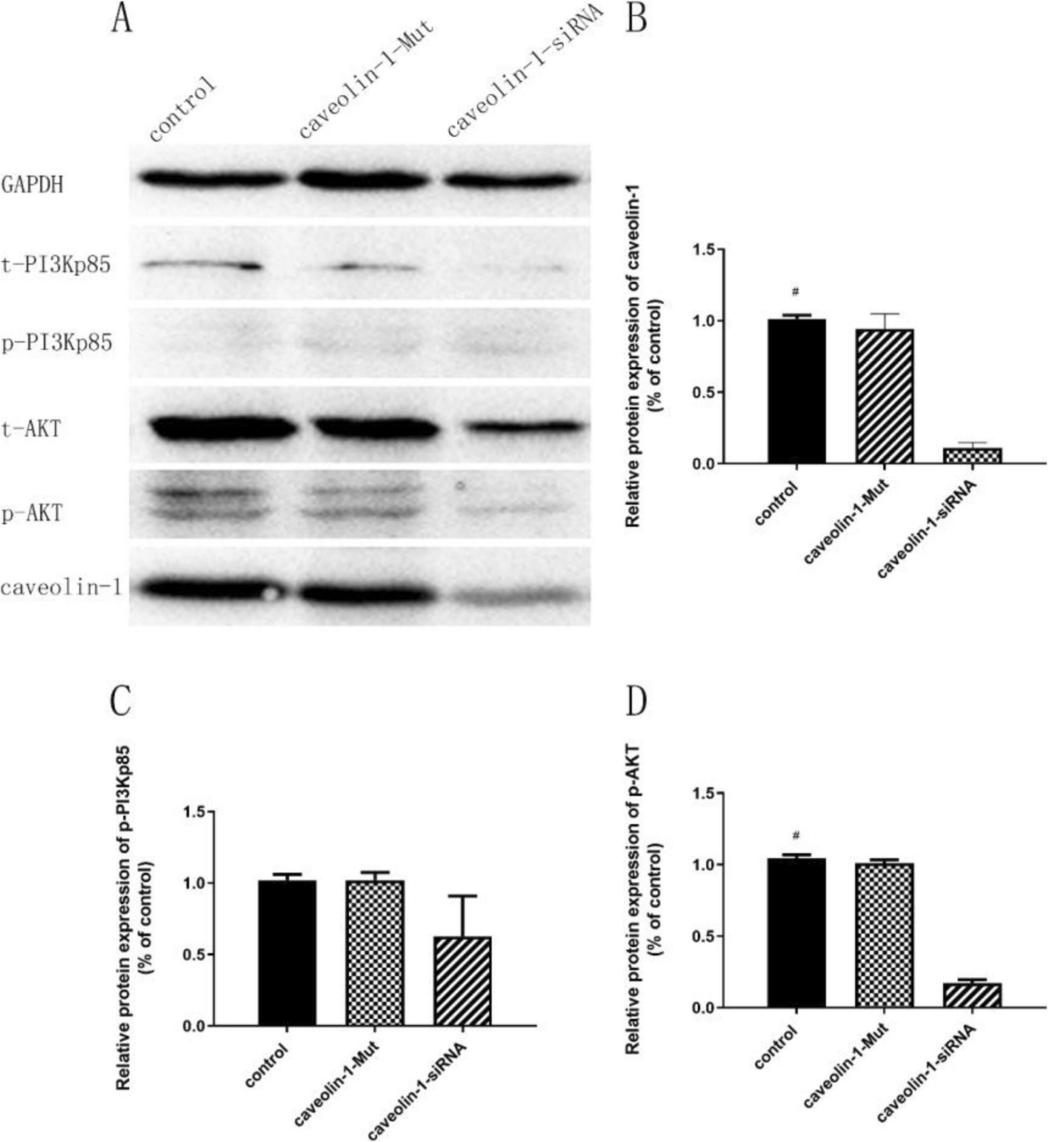
The effect of caveolin-1 knockdown on the caveolin-1 and PI3K/AKT proteins. **a** The protein bands. **b** Comparison of the caveolin-1 concentrations. **c** Comparison of the p-PI3Kp85 concentrations. **d** Comparison of the p-AKT concentrations. p-PI3K, phosphorylated PI3K; p-AKT, phosphorylated AKT; ^#^*P* < 0.05 vs the caveolin-1 siRNA group

Figure 7 showed that the AT (vs control: 95% CI − 2.17 to approximately − 1.28, *P* < 0.001), RT (vs control: 95% CI − 2.23 to approximately − 1.34, *P* < 0.001) and AT+RT (vs control: 95% CI − 2.33 to approximately − 1.44, *P* < 0.001) groups exhibited significant increases in caveolin-1. There was a clear difference in the levels of p-PI3K (95% CI − 0.79 to approximately − 0.09, *P* = 0.012) and p-AKT (95% CI − 0.72 to approximately − 0.03, *P* = 0.030) between the AT+RT and control groups. To elucidate the mechanism by which AT+RT improved EPC function, we investigated the effects of caveolin-1 knockdown on EPC p-PI3K and p-AKT protein expression. AT+RT contributed to a notable increase in the EPC p-PI3K and p-AKT protein levels, which were reduced in caveolin-1 knockdown EPCs (see Fig. 8 for details).

## Discussion

This study explored the effect of 14-day RT, AT and combination training on the function of EPCs and the potential mechanism in mice with type 2 diabetes.

The data revealed that (i) RT, AT and a combination of both can significantly improve the proliferative functions of EPCs in mice with type 2 diabetes and that AT+RT induced the greatest improvement in EPC adherence; (ii) AT+RT induced increases in caveolin-1 and PI3K AKT; and (iii) knockdown of caveolin-1 abolished the positive effect of AT+RT on the functions of EPCs and protein expression (caveolin-1, PI3K, AKT).

Diabetes impairs the angiogenic ability of EPCs, which significantly enhances the risk of atherosclerotic disease and increases cardiovascular morbidity and mortality [2]. AT has been well studied and shown to enhance the function of EPCs in patients with hypertension, obesity, metabolic syndrome, coronary artery disease and chronic heart failure [3–7]. However, the effect of AT, RT or AT+RT on the function of EPCs in people or animal models with type 2 diabetes has not yet been explored.

Our data suggest that exercise (AT, RT and AT+RT) inhibited EPC proliferation. A single session of RT was reported to stimulate SDF-1α, VEGF, hypoxia-inducible factor 1-α (HIF-1α) and matrix metalloproteinase-9 [10–12], which were shown to enhance the proliferative, migratory and angiogenic abilities of EPCs [20–22]. These studies support our finding that RT enhanced EPC proliferative activity in mice with type 2 diabetes. RT or RT combined with AT was documented to increase the number of EPCs in healthy volunteers [10–13]. Our study indicated that an increase in EPC number may contribute to the enhanced EPC proliferative capability after RT or AT+RT.

The positive effects of AT or RT were combined in AT+RT. Mice in the AT+RT group showed especially notable EPC adherence. The AT+RT intervention resulted in the greatest improvements in blood glucose, plasma lipids and fasting insulin in patients with and animal models of type 2 diabetes [23]. Previous studies reported the effect of AT, RT and AT+RT on insulin resistance (HOMA-IR) in patients with high blood glucose, in which the AT+RT resulted in the maximum improvement of HOMA-IR [24], thus alleviating the negative effects of high blood glucose on EPCs. Decreased numbers and dysfunction of EPCs have been shown to be independent risk factors for future cardiovascular events and overall mortality in different populations. Through reversing the diabetes-mediated dysfunction of EPCs, combined aerobic and resistance training may be a possible strategy for the prevention of diabetes-related cardiovascular diseases.

We observed an increase in the protein expression of caveolin-1 after exercise. To further elucidate the mechanism underlying exercise-induced improvement of EPC proliferation and adherence, we explored the impact of caveolin-1 on EPC function by cellular transfection of a lentiviral vector with caveolin-1-siRNA. We found that reduced expression of caveolin-1 and inhibition of EPC functions were detected in EPCs infected with caveolin-1-siRNA. Caveolin-1 is involved in the regulation of transcytosis, permeability, vascular tone and angiogenesis. One study showed that knockdown of caveolin-1 inhibited endothelial cell function [25]. A previous study suggested that treadmill exercise can enhance EPC function via a significant increase in the concentration of caveolin-1 [26]. Consistent with these two studies, our study indicated that AT+RT enhanced EPC function by upregulating caveolin-1 in mice with type 2 diabetes.

Our study showed that exercise promoted EPC function and increased p-PI3K and p-AKT, which were abolished by knockdown of caveolin-1. PI3K/AKT was reported to promote EPC function. Another study demonstrated that the increase in PI3K/AKT induced by exercise can lead to further enhancement of the function of EPCs [27], which was consistent with our study. A previous report demonstrated that the modulation of caveolin-1 expression can affect signalling through the PI3K/AKT pathway and cellular proliferation [28]. AKT expression was downregulated in embryonic fibroblast cells in caveolin-1 knockout mice, resulting in inhibited cell functions [29], which was consistent with our study.

Caveolin-1 has been investigated by a series of studies providing insight into the confusing effects of caveolin-1 on cellular function. In an article published in *Circulation*, we found that endothelial cell function was significantly abrogated in mice without the caveolin-1 gene [30]. Another study also provided similar evidence: caveolin-1 knockdown via caveolin-1 siRNA was reported to decrease p-AKT, resulting in reduced cell proliferation [31]. These two studies provide support for the results of our study. When AKT was activated, p-AKT colocalized with caveolin-1 in human bone marrow-derived mesenchymal stem cells exhibiting osteogenic differentiation [32]. Based on these findings and previous studies of caveolin-1, we hypothesized that the exercise induces alterations invascular shear stress by increasing blood flow and tissue fluid flow [20, 33], which rises the level of vascular endothelial growth factor [16] to trigger an increase of phosphorylated PI3K/AKT by caveolin1 [31, 34]. Caveolin1 through compartmentalization of VEGF receptor with VEGF provides a structural platform to facilitate and amplifies signalling cascades like the PI3K/AKT pathway [31, 35], resulting in increasing the bioactivities of adhesion and proliferation of EPC [16, 35].

The limitations of this study include the fact that in vitro observations cannot indicate the impact on the entire body, the short time period of the intervention and the lack of baseline variables regarding EPC function. However, a control group with no specific exercise intervention was used to identify the effects of exercise and remaining sedentary on EPC function in this randomized controlled study.

## Conclusion

Our study explores the effect of 14-day RT, AT and combination training on the function of EPCs and the potential mechanism in mice with type 2 diabetes. The data revealed that AT+RT induced the greatest improvement in adherence of EPCs and increases in caveolin-1 and PI3K AKT; knockdown of caveolin-1 abolished the positive effect of AT+RT on the functions of EPCs and protein expression (caveolin-1, PI3K AKT). Our study will provide a helpful reference for the selection of exercise methods and promote further development of novel cell-based therapeutic strategies, resulting in improved management of diabetes-related cardiovascular diseases.

## Data Availability

The datasets analysed during the current study are available from the corresponding authors on reasonable request.

## Abbreviations

AT: Aerobic training
RT: Resistance training
AT+RT: Combined aerobic and resistance training
EPCs: Endothelial progenitor cells
Caveolin-1-siRNA: Caveolin-1 small interfering RNA
SDF-1: Stromal cell-derived factor 1
PBS: Phosphate-buffered saline
EBM-2: Endothelial cell basal medium-2
FBS: Foetal bovine serum
VEGF: Vascular endothelial growth factor
FPP: Fibronectin purified protein
FITC: Fluorescein isothiocyanate
UEA: *Ulex europaeus* agglutinin
DiI: Tetramethylindocarbocyanine
ac-LDL: Acetylated low-density lipoprotein
DAPI: 4′,6-Diamidino-2-phenylindole
OD: Optical density
t-p-PI3Kp85: Total PI3K p85
p-PI3Kp85: Phosphorylated PI3K p85
t-AKT: Total AKT
p-AKT: Phosphorylated AKT
HIF-1α: Hypoxia-inducible factor 1-α
HOMA-IR: Homeostasis model assessment insulin resistance
MOI: Multiplicity of infection
