# Correction: Aerobic and resistance training enhances endothelial progenitor cell function via upregulation of caveolin-1 in mice with type 2 diabetes

**DOI:** 10.1186/s13287-022-03194-3

**Published:** 2022-11-07

**Authors:** Lu Zhai, Yuhua Liu, Wenpiao Zhao, Qingyun Chen, Tao Guo, Wei Wei, Zhuchun Luo, Yanfeng Huang, Cui Ma, Feng Huang, Xia Dai

**Affiliations:** 1 Department of Endocrinology, The First Affiliated Hospital of Guangxi Medical University, Nanning, 530021 China; 2 Department of Nursing, Guangxi JiangBin Hospital, Nanning, 530021 China; 3 Department of Cardiology, The First Affiliated Hospital of Guangxi Medical University, Nanning, 530021 China; 4 Department of Gastroenterology, The First Affiliated Hospital of Guangxi Medical University, Nanning, 530021 China; 5 Department of Internal Medicine, The First Affiliated Hospital of Guangxi Medical University, Nanning, 530021 China

## Correction to: Stem Cell Research & Therapy (2020) 11:10 10.1186/s13287-019-1527-z

Following publication of the original article [1], the authors noticed the bar chart of relative protein expression of Caveolin-1 and p-PI3Kp85 is similar in Fig. 7B and C. They had mistakenly duplicated the same statistical result data of p-PI3Kp85 when using GraphadPrim software to export the bar graph of Caveolin-1.

The corrected Fig. 7 is given in this article.

**Fig. 7:**
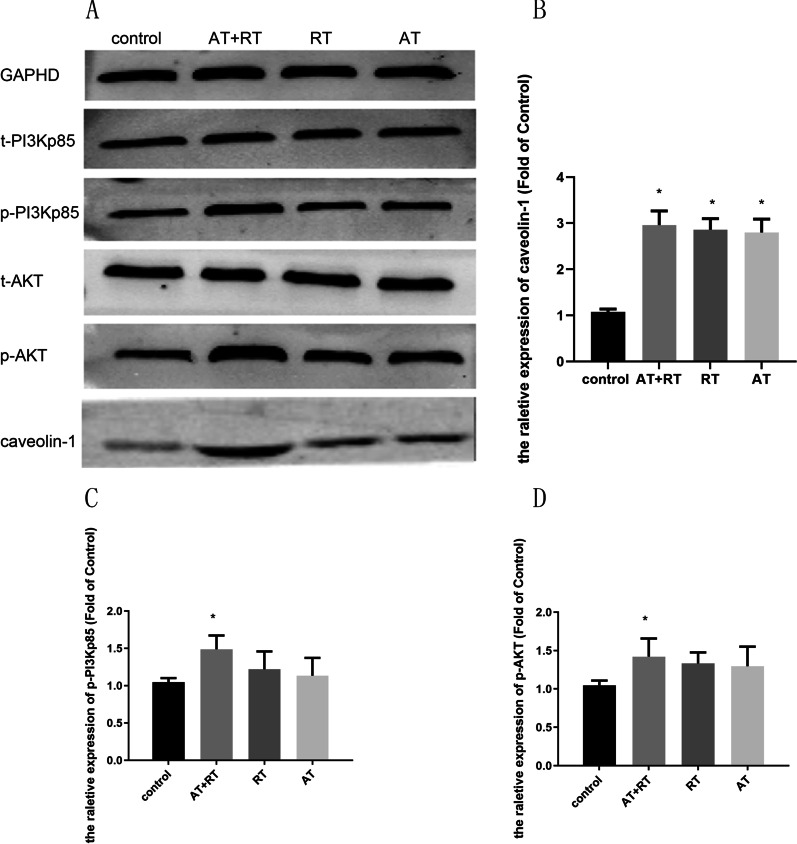
The caveolin-1 and PI3K/AKT protein levels in all groups after intervention. **A** The protein bands after 14 days of intervention. **B** Comparison of the caveolin-1 concentrations among the four groups. **C** Comparison of the p-PI3Kp85 concentrations among the four groups. **D** Comparison of the p-AKT concentrations among the four groups. p-PI3K, phosphorylated PI3K; p-AKT, phosphorylated AKT; AT, aerobic training; RT, resistance training; AT + RT, combination of aerobic and resistance training. *P < 0.05 vs the control group
